# Solid fermentation of wheat bran for hydrolytic enzymes production and saccharification content by a local isolate *Bacillus megatherium*

**DOI:** 10.1186/1472-6750-14-29

**Published:** 2014-04-24

**Authors:** Reda M El-Shishtawy, Saleh A Mohamed, Abdullah M Asiri, Abu-bakr M Gomaa, Ibrahim H Ibrahim, Hasan A Al-Talhi

**Affiliations:** 1 Chemistry Department, Faculty of Science, King Abdulaziz University, P.O. Box 80203, Jeddah 21589, Saudi Arabia; 2 Biochemistry Department, Faculty of Science, King Abdulaziz University, Jeddah, Kingdom of Saudi Arabia; 3 The Center of Excellence for Advanced Materials Research, King Abdulaziz University, Jeddah 21589, Saudi Arabia; 4 Biology Department, Faculty of Science, King Abdulaziz University, Jeddah, Kingdom of Saudi Arabia

**Keywords:** *Bacillus megatherium*, Enzymes, Saccharification, Solid fermentation

## Abstract

**Back ground:**

For enzyme production, the costs of solid state fermentation (SSF) techniques were lower and the production higher than submerged cultures. A large number of fungal species was known to grow well on moist substrates, whereas many bacteria were unable to grow under this condition. Therefore, the aim of this study was to isolate a highly efficient strain of *Bacillus* sp utilizing wheat bran in SSF and optimizing the enzyme production and soluble carbohydrates.

**Results:**

A local strain *Bacillus megatherium* was isolated from dung sheep. The maximum production of pectinase, xylanase and α-amylase, and saccharification content (total soluble carbohydrates and reducing sugars) were obtained by application of the *B. megatherium* in SSF using wheat bran as compared to grasses, palm leaves and date seeds. All enzymes and saccharification content exhibited their maximum production during 12–24 h, at the range of 40–80% moisture content of wheat bran, temperature 37-45°C and pH 5–8. An ascending repression of pectinase production was observed by carbon supplements of lactose, glucose, maltose, sucrose and starch, respectively. All carbon supplements improved the production of xylanase and α-amylase, except of lactose decreased α-amylase production. A little increase in the yield of total reducing sugars was detected for all carbon supplements. Among the nitrogen sources, yeast extract induced a significant repression to all enzyme productivity. Sodium nitrate, urea and ammonium chloride enhanced the production of xylanase, α-amylase and pectinase, respectively. Yeast extract, urea, ammonium sulphate and ammonium chloride enhanced the productivity of reducing sugars.

**Conclusions:**

The optimization of enzyme production and sccharification content by *B. megatherium* in SSF required only adjustment of incubation period and temperature, moisture content and initial pH. Wheat bran supplied enough nutrients without any need for addition of supplements of carbon and nitrogen sources.

## Background

Agricultural residues have an enormous potential as renewable carbon and energy sources. The main potential applications of agricultural residues are in food, animal feed, biofuel and pharmaceutical industries. Saccharification of agricultural residues by microbial hydrolytic enzymes (cellulases, xylanases, amylases and pectinases) is the first step of bioconversion of organic material into reducing sugars, like glucose and xylose [1]. In the saccharification of agricultural residues, a potential effect was detected in presence of two or more enzymes [2]. Cellulases for cellulose hydrolysis [2], xylanases for hemicelluloses hydrolysis [3], amylase for amylose hydrolysis [4] and pectinase for pectin hydrolysis [5] are cooperatively needed in the saccharification of agricultural residues. The reducing sugars obtained from these hydrolyzing actions could be utilized as carbon and energy sources in the fermentation industry, such as lactic acid [6], hydrogen [7] and ethanol [8]. In addition, microbial hydrolytic enzymes utilized in several applications, such as food, textile, paper, pulp and detergent industries [9-12].

Solid state fermentation (SSF) is the growth of organisms on moist substrates in the absence of free-flowing water. The use of SSF for production of enzymes and other products has many advantages over submerged fermentation [13]. These advantages included: easier recovery of products, the absence of foam formation and smaller reactor volumes. Moreover, contamination risks are significantly reduced due to the low water contents and, consequently, the volume of effluents decreases. Another very important advantage is that, it permits the use of agricultural and agro-industrial residues as substrates which are converted into products with high commercial value like secondary metabolites [13,14]. Furthermore, the utilization of these compounds helps in solving pollution problems, which otherwise cause their disposal [15]. For enzyme production, the costs of these techniques are lower and the production is higher than submerged cultures [16,17]. A large number of fungal species was known to grow well on moist substrates in the absence of free-flowing water, whereas many bacteria are unable to grow under this condition [18-21]. As a result, most studies involving SSF have been conducted by using fungi. However, there are little reports of bacterial strains being used successfully for the production of enzymes by using SSF [4,5,22,23]. Therefore, the aim of this study is to isolate strain of *Bacillus* sp. capable of using wheat bran in SSF to produce α-amylase, xylanase and pectinase. The saccharification content, total soluble carbohydrates and reducing sugars, of wheat bran was studied. Studies on optimizing production of enzymes and saccharification content were also carried out.

## Methods

### Isolation, identification and efficiency of the cellulose decomposing bacilli

Five isolates of *Bacillus* spp. were isolated from different samples i.e., sheep dung, horses waste, manure compost and rhizosphere soil. The efficiency of the five strains in cellulose decomposion was estimated using caboxymethyl cellulose (CMC) agar medium containing g/l: CMC, 5; peptone, 5; NaCl, 5; beef extract, 3; agar, 18 and pH was adjusted to 7 [24]. The most efficient strain in cellulose decomposion was identified according to Bergey’s Manual of Systematic Bacteriology [25]. The highest efficient strain in cellulose decomposion was isolated from sheep dung and identified as *B. megatherium*.

### Agricultural residues

Four dried agricultural residues, i.e. wheat bran, date seeds, grass and palm leaves were used as substrates for solid state fermentation (SSF).

### Physicochemical parameters of SSF

Physicochemical parameters of SSF were studied for optimization production conditions of soluble carbohydrates, reducing sugars, α-amylase, pectinase and xylanase by *B. megatherium*. The agricultural residues were sperately sterilized in an autoclave for 20 min at 121°C. *B. megatherium* was grown in 50 ml Erlenmeyer flask included 5 g of the respective sterilized agricultural residue and appropriate amount of water needed to adjust the moisture of dried substrate, which contained 10% moisture after dring. Optimized physicochemical parameters including: incubation period, incubation temperature, and moisture content of the substrate and incubation pH. The pH was adjusted using 0.1 M NaOH or HCl. The influence of supplementation of carbon sources (glucose, maltose, starch, sucrose, and lactose at 1% w/v) and nitrogen sources (yeast extract, urea, sodium nitrate, ammonium sulphate, and ammonium chloride at 1% w/v) has been studied. Each experiment was done in triplicate.

### Soluble carbohydrate and enzyme extraction

Soluble carbohydrate and enzyme were extracted by mixing the fermented substrate with 50 ml distilled water and shaked on a rotary shaker at 180 rpm overnight. The suspension was then centrifuged at 12000 rpm for 10 min and the supernatant was designated as a crude extract.

### Determination of total reducing sugars

Total reducing sugars were determined by the method of Miller [26]. The reaction mixture contained 0.5 ml of crude extract and 0.5 ml dinitrosalicylic acid reagent. The tubes were heated in a boiling water bath for 10 min. After cooling to room temperature, the absorbance was measured at 560 nm. Glucose served as the calibration standard for total reducing sugar determination.

### Determination of total soluble carbohydrates

Total soluble carbohydrates were determined by the method of Dubois et al. [27]. The reaction mixture contained 25 μl of a 4:1 mixture of phenol and water, 0.8 ml of crude extract and 2 ml of concentrated sulfuric acid. Then mixed well, and heated in a boiling water bath for 30 min. The absorbance was determined at 480 nm. Glucose served as the calibration standard for total carbohydrate determination.

### Enzymes assays

α-Amylase, pectinase and xylanase activities were assayed by determining the liberated reducing end products using maltose, galacturonic acid and xylose as standards, respectively [26]. Substrates used were starch, polygalacturonic acid and birchwood xylan for α-amylase, pectinase and xylanase, respectively. The reaction mixture (0.5 ml) contained 1% substrate, 0.05 M sodium acetate buffer pH 5.5 and 0.1 ml crude extract. Assays were carried out at 37°C for 1 h. Then 0.5 ml dinitrosalicylic acid reagent was added to each tube. Then the reaction mixture was mixed well, and heated in a boiling water bath for 10 min. After cooling to room temperature, the absorbance was measured at 560 nm. One unit of enzyme activity is defined as the amount of enzyme which liberated one μmol of reducing sugar per min under standard assay conditions.

All the experimental work was run in triplicates.

### Statistical analysis

The obtained data were statistically analyzed as a randomized complete block design with three replicates by analysis of variance (ANOVA) using the statistical package software SAS (SAS Institute Inc., 2000, Cary, NC., USA). Comparisons between means were made by *F-*test and the least significant differences (LSD) at level *P =* 0.05. Correlations coefficient among the different parameters were also calculated by SAS.

## Results and discussion

### The effect of agricultural residues

**Figure 1:**
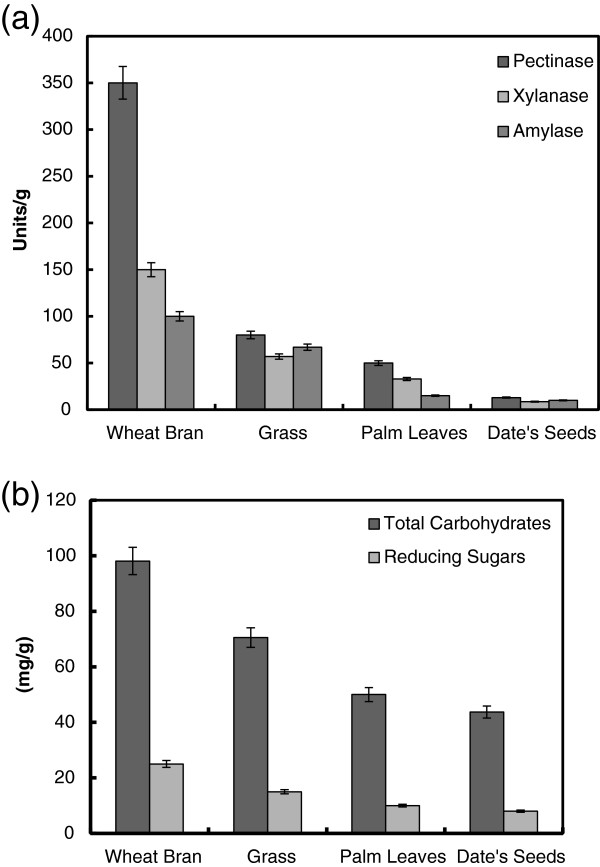
**Effect of different agriculture wastes on the production of pectinase, xylanase and α-amylase (a) and total carbohydrate and reducing sugars (b) by *B. megatherium *in SSF.** Process conditions: incubation time 48 h., initial moisture content 50% (by volume per mass) and temperature at 37°C.

Production of pectinase, xylanase and α-amylase, and saccharification content (total soluble carbohydrates and reducing sugars) by *B. megatherium* were tested in different SSF sources using wheat bran, grasses, palm leaves and date seeds. Figure 1a showed the maximum production of pectinase, xylanase and α-amylase by *B. megatherium* (350, 150 and 100 units/g solid, respectively) in SSF using wheat bran as compared to other agriculture residues. The saccharification content of these agricultural residues improved these results, whereas the productivity of total soluble carbohydrates and reducing sugars exhibited maximum production by using wheat bran (Figure 1b). Similarly, among the lignocellulosic substrates tested, wheat bran gave maximum yield of xylanase by *Cellulosimicrobium* sp. as compared to other substrates viz. gram bran, rice husk, rice bran, wood dust and apple pomace [28]. The best substrate for xylanase production was wheat bran due to its nutritional content and large surface area [29], where wheat bran contained xylan and protein, which were served as carbon and nitrogen sources for microorganisms, respectively [30]. α-Amylase was also exhibited the maximum production by *Clostridium thermosulforegenes* using wheat bran [31]. Wheat bran characterized by its better air circulation, loose particle binding and efficient penetration by mycelia and cheaper, therefore it showed a better prospect economically in fermentation processes [32]. Therefore, physicochemical parameters of SSF using wheat bran for optimization production of enzymes and saccharification content of *B. megatherium* was performed in the following study.

### The effect of incubation period

**Figure 2:**
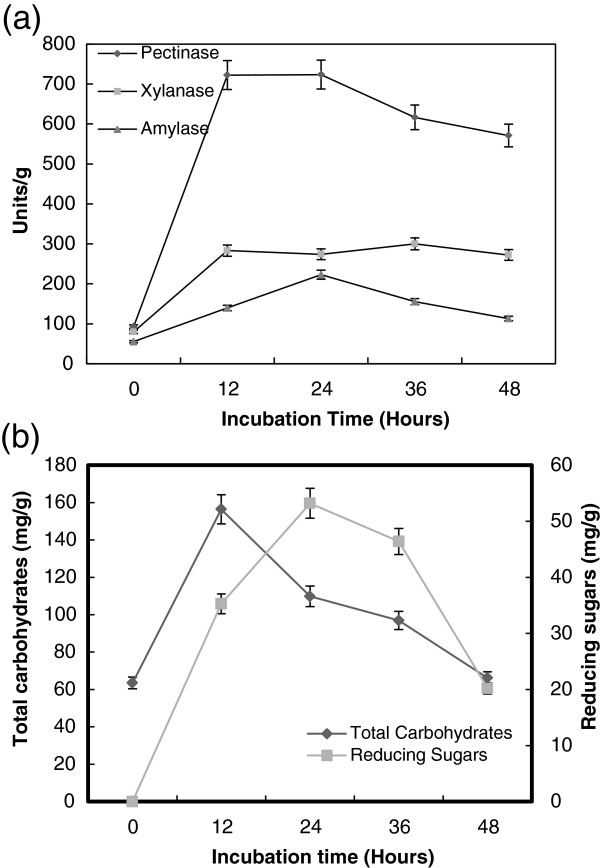
**Effect of incubation time on the production of pectinase, xylanase and α-amylase (a), and total carbohydrate and reducing sugars (b) by *B. megatherium *in SSF using wheat bran as substrate.** Process conditions: initial moisture content 50% (by volume per mass) and temperature at 37°C.

Figure 2a showed the effect of incubation period on production of pectinase, α-amylase and xylanase by *B. megatherium* grown on wheat bran in SSF. Pectinase, α-amylase and xylanase exhibited their maximum production after 24 h (720, 220 and 280 units/g solid, respectively). The maximum production of total soluble carbohydrates (160 mg/g solid) and reducing sugars (55 mg/g solid) was detected after 12 and 24 h, respectively (Figure 2b). The maximum production of reducing sugars was inagreement with the maximum production of enzymes. Similarly, the maximum activity of α-amylase was noted in enzyme extracts harvested after 24 h of SSF *B. subtilis*[33]. The formation of xylanase started from 18 h and reached its maximum after 72 h by *Bacillus* sp. [34]. *B. licheniformis* produced maximum of pectinase after 48 h [5].

### The effect of initial moisture content

**Figure 3:**
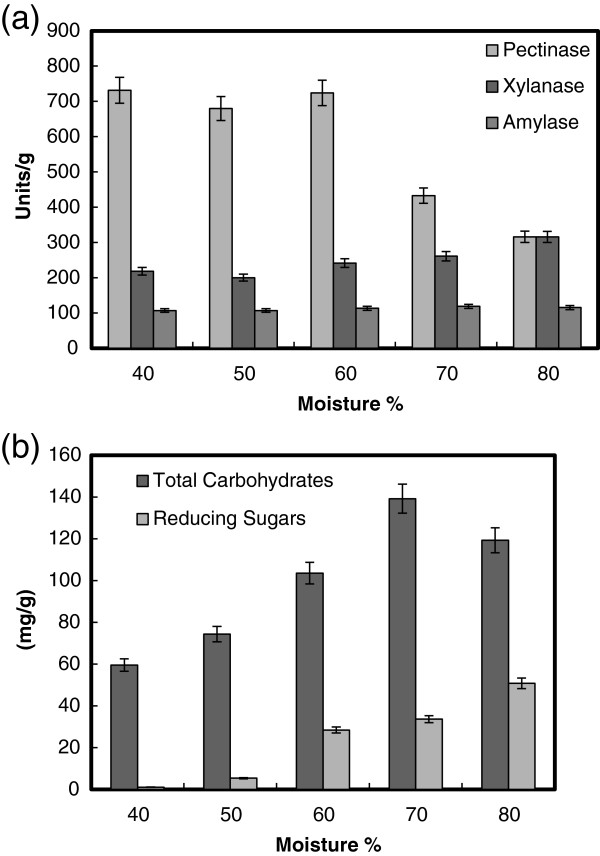
**Effect of moisture % on the production of pectinase, xylanase and α-amylase (a), and total carbohydrate and reducing sugars (b) by *B. megatherium *in SSF using wheat bran as substrate.** Process conditions: incubation times 48 h and temperature at 37°C.

In SSF, the moisture content is an important factor that influences the growth and product yield of microbes [35]. Moisture is reported to cause swelling of the substrates, thereby facilitating better utilization of the substrate by microorganisms [36,37]. The data presented in t Figure 3a, clearly indicated a maximum production of the enzymes pectinase (720 units/g solid), xylanase (310 units/g solid) and α-amylase (115 units/g solid) by *B. megatherium* at 40, 80 and 70% moisture, respectively. In addition, the maximum saccharification content of total soluble carbohydrates (140 mg/g solid) and reducing sugars (55 mg/g solid) was observed at 70 and 80% moisture, respectively (Figure 3b). Other results showed that the yield of α-amylase and amylopullulanase by *Clostridium thermosulfurogenes* increased with an increase in solid to moistening agent ratio from 1:0.5 to 1:2.5 (30 to 75%) with a maximum at 1:2.25 (73%) [31]. Optimum xylanase production from *Bacillus* sp. PKD-9 was observed when wheat bran was used at 1:4 ratio of solid substrate-to-moisture [23]. Any further increase in the ratio resulted in the decrease of enzyme yields may be due to clumping of solid particles which results in the decrease of interparticle space and diffusion of nutrients [29,37]. In contrast, the low moisture content leads to the decreased solubility of nutrients present in the wheat bran thereby decreased enzyme yields [38].

### The effect of incubation temperature

**Figure 4:**
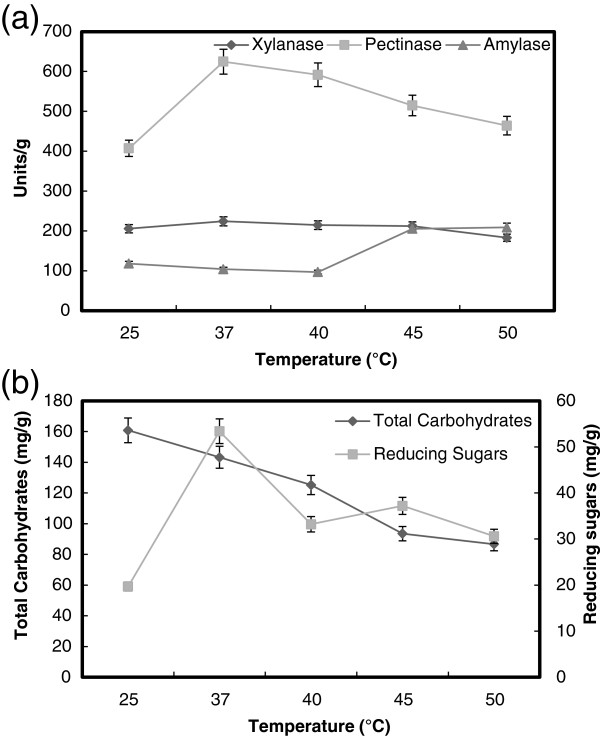
**Effect of incubation temperature on the production of pectinase, xylanase and α-amylase (a), and total carbohydrate and reducing sugars (b) by *B. megatherium *in SSF using wheat bran as substrate.** Process conditions: incubation times 48 h and initial moisture content 50% (by volume per mass).

The usual temperature maintained in SSF systems is in the range of 25-32°C, depending on the growth kinetics of microorganism employed for fermentation purposes [39]. In the present study, the maximum production of both pectinase (620 units/g solid) and xylanase (220 units/g solid) by *B. megatherium* was achieved at 37°C, and 45°C for α-amylase (220 units/g solid) (Figure 4a). These results were confirmed by maximum saccharification content of reducing sugars (54 mg/g solid) at 37°C, where the maximum of total soluble carbohydrates (160 mg/g solid) was detected at 25°C (Figure 4b). In other study, the optimum temperature recorded for maximum growth and α-amylase production by *B. subtilis* was 35°C [33]. Maximum production of pectinase and xylanase were obtained by *B. licheniformis*[5] and *Bacillus* sp. PKD-9 [23] at 37°C in solid wheat bran.

### The effect of pH

**Figure 5:**
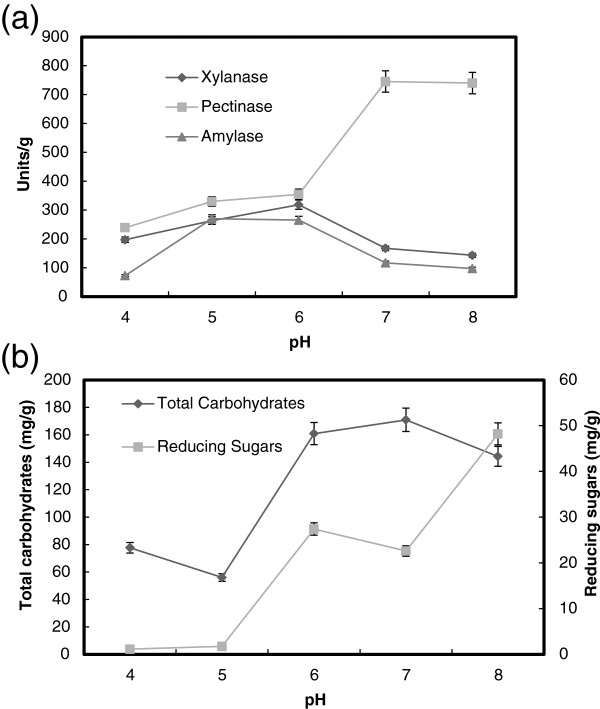
**Effect of pH on the production of pectinase, xylanase and α-amylase (a), and total carbohydrate and reducing sugars (b) by *B. megatherium *in SSF using wheat bran as substrates.** Process conditions: incubation times 48 h, initial moisture content 50% (by volume per mass) and temperature at 37°C.

The initial pH of fermentation medium has a significant effect on bacterial growth and enzyme production [40]. In order to study the effect of pH on enzyme production and saccharification content of wheat bran by *B. megatherium*, the production medium having pH's ranged from 4 to 8. Figure 5a shows a maximum production of pectinase (750 units/g solid), xylanase (280 units/g solid) and α-amylase (250 units/g solid) by *B. megatherium* at pH 7, 6 and 5, respectively. The high activity of pectinase results was reinforced by maximum saccharification content of total soluble carbohydrates (170 mg/g solid) and reducing sugars (50 mg/g solid) and at pH 7–8 (Figure 5b). However, most of *Bacillus* species were found to be produce maximum pectinase at different pH's ranging from 7 to 9 [41]. Further, the type of buffer used in nutrient solution is a key factor in governing α-amylase production by the *B. subtilis*.

### The effect of supplementation carbon and nitrogen sources

**Figure 6:**
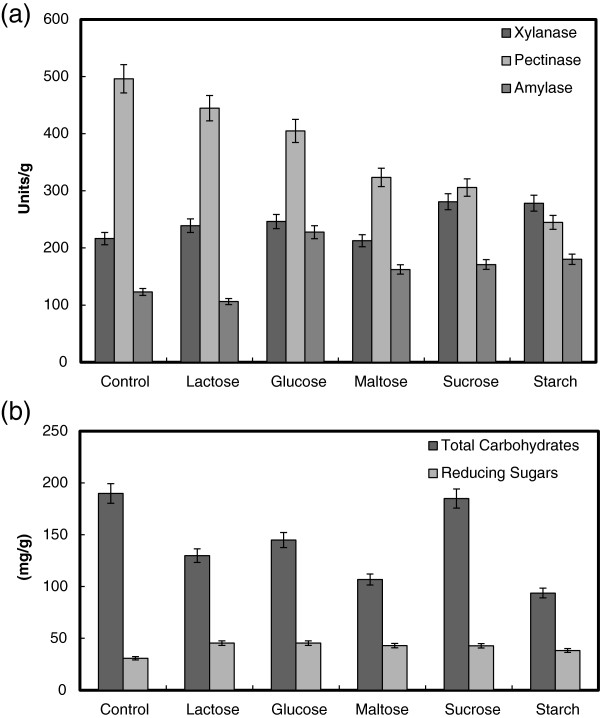
**Effect of carbon source (1%) supplementation on the production of pectinase, xylanase and α-amylase (a), and total carbohydrate and reducing sugars (b) by *B. megatherium *** **in SSF using wheat bran as substrates.**

The influence of supplementary carbon sources such as starch, sucrose, maltose, lactose or glucose at 1% (by mass) to wheat bran on production of pectinase, xylanase and α-amylase by *B. megatherium* was studied (Figure 6a). An ascending inhibition of pectinase production by lactose, glucose, maltose, sucrose and starch was detected, respectively. However, all carbon supplements improved the production of xylanase and α-amylase, except of lactose decreased α-amylase production. While most of carbon supplements exhibited repressive effect on the production of total soluble carbohydrates except of sucrose, a little increase in the yield of total reducing sugars was detected (Figure 6b). The same carbon supplements except starch caused repressive effect on pectinase production by *B. licchenformis*[5]. Supplementation of carbon sources increased α-amylase production by *B. cereus* during SSF using wheat bran [22]. *B. thermooleovorans* is reported to prefer starch, glucose, lactose, maltose and maltodextrins as carbon sources for α-amylase secretion [42,43]. In contrast, carbon sources such as glucose, maltose and starch did not enhance α-amylase production by thermophilic *B. coagulans* in solid-state fermentation using wheat bran [29]. Xylanase production by *Bacillus* sp. AR-009 grown on wheat bran was repressed upon addition of lactose, glucose and sucrose [3].

**Figure 7:**
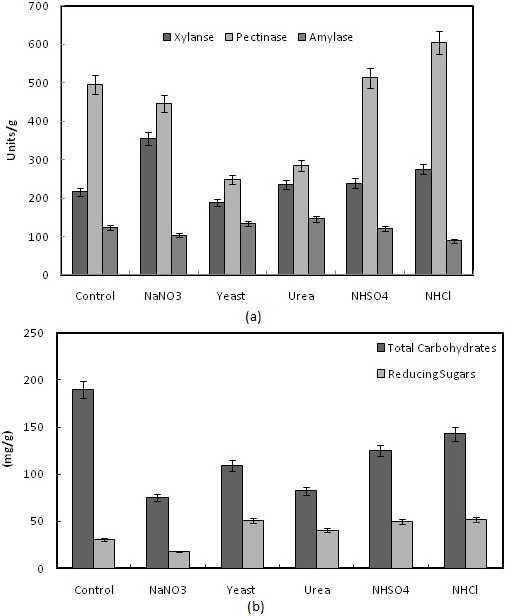
**Effect of nitrogen source (1%) supplementation on the production of pectinase, xylanase and α-amylase (a), and total carbohydrate and reducing sugars (b) by *B. megatherium *in SSF using wheat bran as substrates.** Process conditions: incubation times 48 h, initial moisture content 50% (by volume per mass) at temperatures 37°C.

Studies on supplementation of nitrogen sources such as ammonium sulphate, ammonium nitrate, ammonium chloride, yeast extract or urea at 1% concentration to the wheat bran showed various effects on pectinase, xylanase and amylase production by *B. megatherium* (Figure 7a). Among the nitrogen sources, yeast extract induced a significant repression to all enzyme productivity. While sodium nitrate inhibited α-amylase production from 123 to103 units/g, it enhanced xylanase production from 216 to 355 units/g. Urea on the other hand inhibited production of xylanase and enhanced α-amylase. Also, a significant enhancement of pectinase activity was obtained by ammonium chloride supplementation from 496 to 610 units/g. While all nitrogen sources inhibited the production of total soluble carbohydrates, yeast extract, urea, ammonium sulphate and ammonium chloride enhanced the productivity of the total reducing sugars (Figure 7b). Hashemi et al. [4] reported that ammonium nitrate was the best inducer for α-amylase secretion followed by yeast extract ‘by *Bacillus* sp. On the other hand, the limitation or starvation of nitrogen during the fermentation resulted in the limited growth of *B. subtilis* and the enhancement of α-amylase production [44]. For *Bacillus* sp. AR-009, yeast extract increased xylanase production [3]. Maximum production of pectinase was achieved when yeast extract was used in fermentation medium [5].

## Conclusions

In conclusion, the production of pectinase, xylanase and amylase and saccharification content (total soluble carbohydrates and reducing sugars) by a newly local isolat *B. megatherium* using wheat bran in SSF will have several advantages. The optimization of enzyme production and sccharification content required only adjustment of incubation time and temperature, moisture content and initial pH. Wheat bran supplied enough nutrients without any need for addition of supplements of carbon and nitrogen sources. All these combined together could greatly reduce the overall cost of production of enzymes and saccharification content by *B. megatherium*. In the future, the reducing sugars will be used for hydrogen production.

## Competing interests

The authors declare that they have no competing interests.

## Authors’ contributions

El-R, MS, AA, GM, II and AL-H performed all experiments and read and approved the final manuscript.
